#  Hairy vocal cords and hemoptysis 

**DOI:** 10.5578/tt.202501992

**Published:** 2025-03-24

**Authors:** Nurdan ŞİMSEK VESKE, Sinem Nedime SÖKÜCÜ, Gülşah GÜNLÜOĞLU, Sedat ALTIN

**Affiliations:** 1 Clinic of Chest Disease, University of Health Sciences Yedikule Chest Diseases and Thorasic Surgery Training and Research Hospital, İstanbul, Türkiye

## Abstract

**ABSTRACT**

** Hairy vocal cords and hemoptysis **

* A 71-year-old patient with hemoptysis, history of pulmonary tuberculosis, laryngeal cancer, and smoking habit underwent fiberoptic bronchoscopy. No endobronchial lesions were detected, but hair structures were seen on the vocal cords. Klebsiella oxytoca was grown in the non-specific culture of the lavage. Filamentous bacteria suggestive of actinomyces were also seen in the cytology. These bacteria, seen in the normal flora of the oropharynx, may be responsible for lung infection in individuals with weakened immune systems and concomitant diseases. In elderly patients with a history of pulmonary tuberculosis and cancer, as in our patient, hairs developing on the vocal cords as a complication of laryngeal surgery may cause food residues and secretions to accumulate on the hair surface and increase microaspirations. We would like to remind you that these microaspirations may affect the lower respiratory tract defense mechanisms and lead to clinical problems such as lung infection, lung abscess, aspiration pneumonia and hemoptysis in people with comorbid diseases. *

**Key words:** *Complication; hair; hemoptysis; larynx cancer *

**ÖZ**

** Kıllı vokal kord ve hemoptiz **

* Yetmiş bir yaşında, hemoptizisi bulunan ve özgeçmişinde akciğer tüberkülozu ve larenks kanseri öyküsü bulunan, sigara içme alışkanlığına sahip hastaya fiberoptik bronkoskopi uygulandı. Endobronşiyal lezyon saptanmadı, ancak vokal kordlar üzerinde kıl yapıları izlendi. Lavajda non-spesifik kültürde Klebsiella oxytoca üremesi tespit edildi. Sitolojisinde ise aktinomiçesi düşündüren filamentöz bakteriler gözlendi. Orofarenkste normal florada bulunan bu bakteriler, immün sistemi zayıf ve komorbid hastalığı olan kişilerde pulmoner enfeksiyona yol açabilir. Bizim hastamızdaki gibi geçmişte tüberküloz ve kanser öyküsü bulunan, ileri yaşta olan hastalarda, larenks cerrahisi sonucu vokal kordlarda gelişen kıllar, kıl yüzeyinde biriken yiyecek artıkları ve sekresyonlar ile artan mikroaspirasyonlara neden olabilir. Bu mikroaspirasyonlar, alt solunum yolu savunma mekanizmalarını etkileyerek, komorbid hastalığı olan kişilerde akciğer enfeksiyonu, akciğer apsesi, aspirasyon pnömonisi gibi klinik sorunlara yol açabilir ve bunların sonucu olarak hemoptiziye neden olabilice- ğini belirtmek istiyoruz. *

**Anahtar kelimeler:** *Komplikasyon; kıl; hemoptizi; larenks kanseri *

## INTRODUCTION

Various complications may be seen depending on the structure of the flap used in larynx surgery. Especially in flaps that contain skin surface, hair growth may occur in the reconstructed areas due to hair growth. In the literature, it has been mentioned that patients who experience asthma-like attacks due to hair growth and that microaspirations may also be seen due to food residues and secretion accumulation on them (1). Since the most common cause of hemoptysis is infections, we present our case with the aim of emphasizing the increased risk of pulmonary infection in patients undergoing such surgical intervention, to be a reminder for bronchoscopists when they encounter such a situation, and to emphasize the importance of surgical techniques that otolaryngologists will use to prevent systemic complications that may develop afterwards.

## Case

A 71-year-old male patient who had hemoptysis mixed with sputum was referred to our outpatient

clinic for further examination. His medical history included surgery for laryngeal cancer 20 years ago and a history of pulmonary tuberculosis in 2000. There was no abnormality in his family history. The patient, who had a 20-pack-year smoking history, had not smoked for 35 years. No pathology was detected in the physical examination of the respiratory system and other systems.

In the thorax computerized tomography (CT), there were focal bronchiectatic changes in the apical area of the right lung, focal density increase, and pleural thickening. There were a few millimetric air cysts in the upper lobes of both lungs and pleural nodularities and thickening in the left apical area (Figure 1). His hemogram showed neutrophil-dominant leukocytosis. Other biochemical parameters were normal. Fiberoptic bronchoscopy was planned due to the patient’s age, smoking history, previous pulmonary tuberculosis, and history of laryngeal canncer. Bronchoscopy showed purulent secretions and hair structures at the vocal cord level (Figure 2).

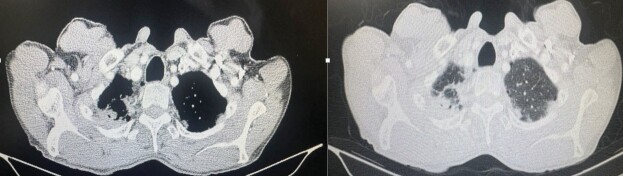

**Figure 1.** Pleural thickening, sequel fibrotic changes.

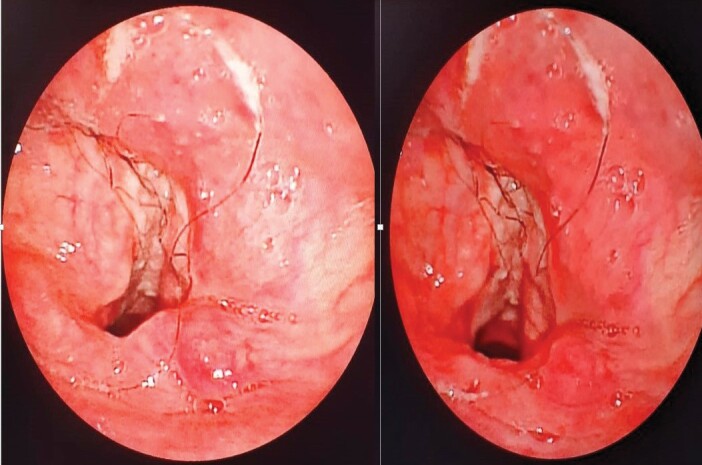

**Figure 2.** Hair growth at the reconstructed site.

**78** Tuberk Toraks 2025;73(1):77-79

Şimsek Veske N, Sökücü SN, Günlüoğlu G, Altın S.

The trachea and both bronchial systems were open. The procedure was terminated by taking lavage from both upper lobes. The patient’s detailed anamnesis indicated that the hair formations in the vocal cords developed after a larynx surgery he had 20 years ago, that he was being followed up by the ear, nose and throat clinic, and that the hairs that had grown were cleaned with laser at certain intervals. Lavage *Mycobacterium tuberculosis* acido-resistant bacilli and polymerase chain reaction were negative. *Klebsiella oxytoca* growth was detected in the non- specific culture of the lavage. Bronchial epithelial cells, alveolar histiocytes, squamous epithelial cells, filamentous bacteria were observed in the lavage cytology, periodic acid schiff was found positive from the applied histochemical stains, and *Actinomyces* was primarily considered, it was reported. Ciprofloxacin and amoxicillin clavulonic acid treatment, which were found to be sensitive according to the lavage culture antibiogram, were started. The hemoptysis of the patient, who did not have previous thorax CT images to compare, resolved and the patient was followed up.

## DISCUSSION

Hemoptysis is defined as coughing up blood originating from the lungs or bronchi. Approximately 60-70% of hemoptysis is due to infections. Inflammation and edema develop due to infection, which causes the mucosal superficial blood vessels to rupture (2). Although actinomycosis and *K. oxytoca* are normal flora in the mouth, oropharynx and intestine, they cause devastating damage to the lungs when aspirated (3). It results in a higher rate of infection in individuals with compromised immune systems. The patient population typically includes individuals with weakened immune response, such as those with diabetes, alcoholism, cancer, liver diseases, chronic obstructive lung disease, and chronic renal failure. The rate of infection is also high in these patient groups. *Klebsiella* can be transmitted from one hospitalized patient to another.

It has been stated in publications that hair growth may occur in the reconstructed area due to the skin surface of the platysma myocutaneous flap and

pectoralis major flaps used as a complication in larynx surgery (4,5). Patients with hairy laryngeal flaps in particular often complain of irritation, voice change, tickling, gagging, difficulty swallowing, food impaction, and bad breath (4). Accumulation of food residues and secretions on the hairs and increased microaspirations may also affect the lower respiratory tract defense mechanisms and cause clinical problems such as lung abscess and aspiration pneumonia in people with comorbid diseases.

As a result, we emphasize the importance of multidisciplinary evaluation of the patient by reminding that hair growth may occur in the vocal cords as a complication of larynx surgery, and that secondary pulmonary pathologies may develop when bronchoscopists encounter such a situation.

## CONFLICT of INTEREST

The authors have no conflict of interest to declare.

## AUTHORSHIP CONTRIBUTIONS

Concept/Design: NŞV Analysis/Interpretation: NŞV Data acqusition: NŞV, GG Writing: NŞV

Clinical Revision: SNS, GG Final Approval: SA

## REFERENCES

1. *Nemerov EV. Asthma-like attacks caused by growth of hair in the skin transplant in the larynx. Klin Med (Mosk) 1982; 60(11): 93-5.*
2. *Weinberger SE, Braunwald E. Cough and hemoptysis. In: Harrison’s Principles od Internal Medicine, 15th ed. New York: McGraw-Hill. 2001: 203-6.*
3. *Ryan KJ, Ray CG. Sherris Medical Microbiology. 4th Edition (2004). McGraw-Hill, New York.*
4. *Toft K, Keller GS, Blackwell KE. Ectopic hair growth after flap reconstruction of the head and neck. Arch Facial Plast Surg 2000; 2(2): 148-50. https://doi.org/10.1001/archfaci.2.2.148*
5. *Min-Woo P, Seung-Kuk B, Kwang-Yoon J. Laser epilation for unwanted hair in the larynx. Med Lasers 2012; 1(1): 31-3. https://doi.org/10.25289/ML.2012.1.1.31*

